# Sparsity-Driven Reconstruction Technique for Microwave/Millimeter-Wave Computational Imaging

**DOI:** 10.3390/s18051536

**Published:** 2018-05-12

**Authors:** Thomas Fromenteze, Cyril Decroze, Sana Abid, Okan Yurduseven

**Affiliations:** 1 XLIM UMR 7252, Université de Limoges/CNRS, 87060 Limoges, France ; cyril.decroze@xlim.fr (C.D.); sana.abid@xlim.fr (S.A.); 2 Jet Propulsion Laboratory, California Institute of Technology, Pasadena, CA 91109, USA; okanyurduseven@ieee.org

**Keywords:** computational imaging, short-range imaging, microwave, millimeter-wave, sparsity

## Abstract

Numerous prototypes of computational imaging systems have recently been presented in the microwave and millimeter-wave domains, enabling the simplification of associated active architectures through the use of radiating cavities and metasurfaces that can multiplex signals encoded in the physical layer. This paper presents a new reconstruction technique leveraging the sparsity of the signals in the time-domain and decomposition of the sensing matrix by support detection, the size of the computational inverse problem being reduced significantly without compromising the image quality.

## 1. Introduction

**Figure 1:**
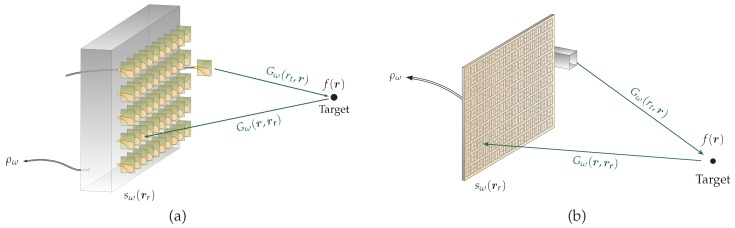
Computational imaging architectures based on the use of a cavity connected to a conventional antenna array (**a**) and the design of a dispersive metasurface (**b**). In each case, the signals reflected by the target are encoded by propagation within the reception system into a measured signal $\rho_{\omega }$.

Microwave and millimeter-wave imaging applications are becoming increasingly numerous and cover a wide range of fields such as medical diagnostics [1,2,3,4], non-destructive testing [5,6,7], and concealed weapon detection [8,9,10]. However, all of these systems, in order to satisfy reasonable acquisition times, are constrained by the implementation of complex and redundant active systems, which represents a major economic constraint on the large-scale development of these applications. Faced with these limitations, a multiplicity of computational solutions have emerged, exploiting the availability of increasingly powerful and affordable digital processing units. These solutions are based on the development of predominantly passive components, capable of encoding and multiplexing radiated information in transmission and reception, thus reducing the amount of active channels required for imaging systems to function properly. The constraint is thus pushed back into the digital layer where the formulation and resolution of inverse problems represent new challenges that can make these solutions competitive. It has been demonstrated that such systems can be based on the use of electrically large cavities connected to conventional antenna arrays [11,12,13], on the use of metasurfaces encoding information directly in the radiating aperture [14,15,16], or even on hybrid solutions of leaky cavities that demonstrate interesting performance in many imaging modalities [17,18,19]. Connections can also be made with earlier systems based on the use of frequency scanning antennas whose radiation patterns can encode a sum of information relative to the position of a target into a reduced number of signals [20,21,22]. It is necessary to study all these systems in order to analyze them by means of a unified formalism taking into account the propagation of signals, their filtering within dispersive components, and their summation, as described in [23] and Figure 1.

Regardless of the passive computational system implemented, the objective is the estimate of the reflection function of the target f(r) considered invariant according to frequency in the operating bandwidth, from a single compressed signal represented here by $\rho_{\omega }$, where ω is the pulsation. As a first approximation, it is possible to consider a scalar propagation model between two arbitrary positions $r_{a}$ and $r_{b}$ represented by free space Green’s functions $G_{\omega }\left(r_{a},r_{b}\right)=\exp(-jk|r_{a}-r_{b}\left|\right)/\left|r_{a}-r_{b}\right|$. The expression of the measured signal as a function of the signature of the target linearized assuming Born’s first approximation is as follows:

(1) $$\begin{array}{c} \rho_{\omega }=\int_{r_{r}}\int_{r}G_{\omega }\left(r_{t},r\right)\;f\left(r\right)\;G_{\omega }\left(r,r_{r}\right)\;d^{3}r\;H_{\omega }\left(r_{r}\right)\;d^{2}r_{r} \end{array}$$

where $r_{r}$ corresponds to the coordinates of the radiating aperture and where $r_{t}$ is the location of the transmitting antenna. The most important element of this formula is the vector $H_{\omega }\left(r_{r}\right)$, which corresponds to the response of the component encoding the received information. In the case of an electrically large cavity connected to an array of isotropic antennas, $H_{\omega }\left(r_{r}\right)$ simply corresponds to the transfer functions of the cavity. In the case of a radiating metasurface, or in the case where it is not possible to neglect the impact of radiating elements connected to a cavity, $H_{\omega }\left(r_{r}\right)$ stands for the near field radiated by this structure, which can be divided into a sum of secondary dipoles interacting with the target. In general terms, the principle of computational imaging applied to the microwave and millimeter-wave domains consists in using structured radiation patterns with a low degree of correlation in order to encode the information contained in the target space into electrical signals measured on a reduced number of ports in order to limit the costs and complexity associated with active systems. In contrast to synthetic aperture systems, which can also satisfy these constraints, computational systems also make it possible to quickly capture scenes to be imaged, making their usage compatible with real-time applications [12]. The most comprehensive approach in this framework consists of formalizing the interaction between measured signals and space to be imaged by means of a matrix operator *M*, giving rise to the expression of the following direct problem:

(2) $$\begin{array}{c} \rho =Mf \end{array}$$

where $\rho \in C^{\;n_{\omega }\times 1}$ and $f\in C^{\;n_{r}\times 1}$ are, respectively, the vectorized measured signal including $n_{\omega }$ frequency samples and the reflection function of the target represented by $n_{r}$ spatial samples (for the sake of clarity, bold notation is used for all the vectors), and $M\in C^{\;n_{\omega }\times n_{r}}$ is the sensing matrix accounting for the forward and backward wave propagations, as well as the response of the computational imaging component, as described in Equation (1). Quite obviously, $n_{\omega }$ corresponds to the number of frequency samples and $n_{r}$ to the number of voxels of the discretized target space. This relation makes it clear that spatial information is encoded in a frequency signal and that the rank of the sensing matrix—directly related to the pseudo-orthogonality between the radiated patterns at each frequency—is the main limitation of the number of unknowns that can be reconstructed. This approach is undoubtedly the simplest and so far the most accurate way of reconstructing an estimation $\hat{f}$ by solving the inverse problem through, for example, a pseudo-inversion:

(3) $$\begin{array}{c} \hat{f}=M^{+}\rho \end{array}$$

or through iterative reconstruction techniques, exploiting for example prior knowledge on the inherent sparsity of the interrogated scene [24,25,26,27]. Although this approach is particularly precise and simple to implement, it can suffer from prohibitive memory consumption and computing time, imposing great constraints on the processing units implemented in this framework [23]. Alternative approaches proposed in previous work explored the possibility of breaking down the M measurement matrix into two operators of reduced dimensions, reconstructing in this context an estimate of the signals in the radiating aperture [11,12,28]. The main advantage of this technique lies in the use of Fourier-based image reconstruction techniques on the estimated signals, exploiting the formidable computational efficiency of fast Fourier transforms [13,29]. Using the previous formalisms, the expression of the signals in the radiating aperture is defined as follows:

(4) $$\begin{array}{c} s_{\omega }\left(r_{r}\right)=\int_{r}G_{\omega }\left(r_{t},r\right)\;f\left(r\right)\;G_{\omega }\left(r,r_{r}\right)\;d^{3}r \end{array}$$

so that the measured signal can be written as follows:

(5) $$\begin{array}{c} \rho_{\omega }=\int_{r_{r}}s_{\omega }\left(r_{r}\right)\;H_{\omega }\left(r_{r}\right)\;d^{2}r_{r}. \end{array}$$

**Figure 2:**
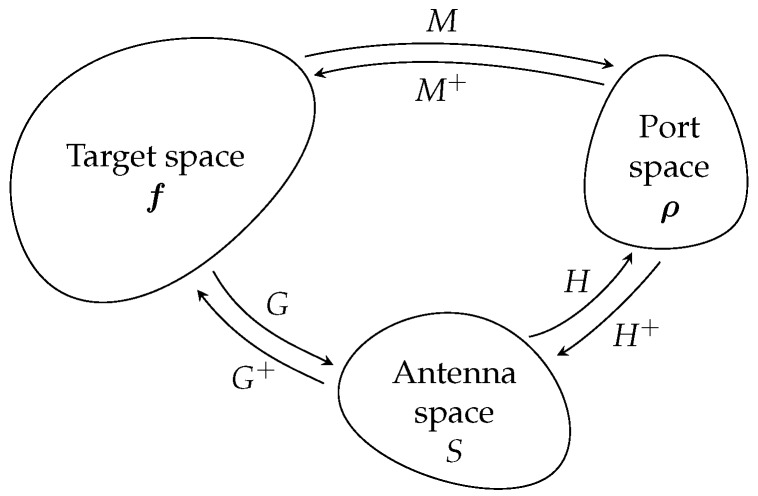
Representation of the subspaces considered for these computational imaging problems. An estimate of the target reflectivity function f is made from the measured signal ρ. The approach studied in this article is based on a decomposition of the operator *M* linking the target to the measurement ports via an estimate of the signals *S* in the radiating aperture.

The reconstruction of the target response can thus be achieved by introducing an intermediate subspace corresponding to that of the signals in the radiating aperture, as shown in Figure 2.

The main limitation related to the current implementation of this technique is illustrated by writing the last equation in matrix form, spatially discretizing the radiating aperture into $n_{r_{r}}$ samples:

(6) $$\begin{array}{c} \rho =\sum_{r_{r}}H⊙S \end{array}$$

where $H\in C^{\;n_{\omega }\times n_{r_{r}}}$ and $S\in C^{\;n_{\omega }\times n_{r_{r}}}$ correspond, respectively, to the transfer functions of the computational imaging component and the signals in its radiating aperture, and where ⊙ stands for the Hadamard (or element-wise) product. Since the signal to be reconstructed $\hat{S}$ has more unknowns than the number of measured samples in ρ, it seems impossible to calculate an accurate estimate of the latter unless the frequency dimension is sacrificed in a pseudo-inversion calculation, which would prevent the backpropagation computation for the estimate of $\hat{f}$. The approach suggested in the literature, initially inspired by time-reversal [11], was to use a simple equalization of the transfer functions of the component using the pre-computed pseudo-inverse $H^{+}$ [12,13]:

(7) $$\begin{array}{cc} {\hat{S}}_{eq} & =H^{+}⊙\;P \end{array}$$

where P=(ρ,…,ρ) is the concatenation of ρ $n_{r_{r}}$ times to match the dimensions of *H*. It is then possible to define an operator $G\in C^{\;n_{\omega }.n_{r_{r}}\times n_{r}}$ taking into account the forward and backward wave propagation, linking the antenna signals with the reflectivity of the target as follows:

(8) $$\begin{array}{c} \mathrm{vec}(S)=Gf \end{array}$$

where $\mathrm{vec}\left(S\right)\in C^{\;n_{\omega }.n_{r_{r}}\times 1}$ is vectorized to match the number of columns of *G*. In this context, it is finally possible to obtain an estimate of $\hat{f}$ from the signals on the antennas reconstructed by equalization using the pseudo-inverse $G^{+}$:

(9) $$\begin{array}{c} \hat{f}=G^{+}\mathrm{vec}\left({\hat{S}}_{eq}\right). \end{array}$$

Although independent signal equalization estimation cannot be ideal due to the ratio of the number of measured points to that of unknowns, the coherence of frequency content during the backpropagation operation represented by $G^{+}$ yields satisfactory results in various applications [12,13]. The distortions observed during the use of this method are counterbalanced by the great gain in calculation time compared to the sensing matrix approach, realized by reducing the dimensions of the problem and replacing the matrix operator $G^{+}$ by range migration algorithms based on fast Fourier transforms [23].

Based on all these elements, a technique based on an identical decomposition of the sensing matrix *M*, compatible with the use of Fourier techniques for backpropagation but allowing more accurate reconstructions of the signals in the radiating aperture, is proposed and studied here. The theoretical principle, based on the exploitation of sparsity in the time-domain, is presented in the next section, which is followed by theoretical and experimental studies.

## 2. Theoretical Principle of a Sparsity-Based Time-Domain Signal Estimation

**Figure 3:**
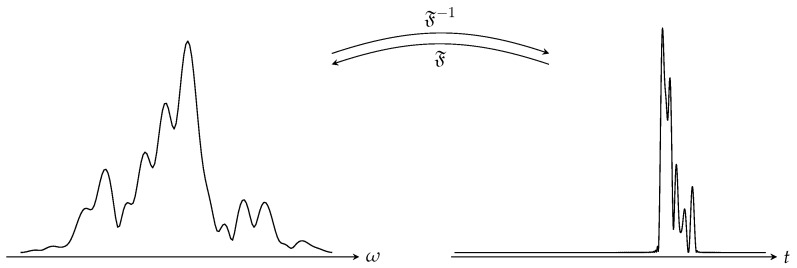
Sparsity of the magnitude of a frequency signal collected in a short range imaging application and its time-domain representation calculated from an inverse Fourier transform $F^{-1}$. An approximate knowledge of the depth spread of the area to be imaged allows for the direct selection of useful samples in the time domain.

All short-range imaging applications mentioned in the first part of the introduction of this document share interesting characteristics, which are directly exploited by the proposed technique: the targets studied have a limited depth extension and are interrogated with ultra-wideband signals. These properties have the advantage of representing signals collected by the antennas from the target in a limited number of points compared to the frequency domain where the corresponding phase correction and subsampling operations are less intuitive and direct, as shown in Figure 3.

This attribute partly responds to the problem of reconstructing space-frequency signals in the radiating aperture from a limited number of measured frequency information. The second constraint that needs to be addressed is the replacement of this extremely simple yet inefficient equalization operation with a matrix approach that can be solved by means of various pseudo-inversion techniques, which may be direct such as the Tikhonov regularization [12] or the truncated singular value decomposition [23], or iterative such as least squares-based techniques [16,30] or the generalized minimal residual method [17,31]. Using the sparsity properties illustrated above, this reconstruction is carried out in the time domain, working on the expression of the measured signal according to those received in the radiating aperture, filtered by propagation in the computational imaging component. Equation (6) takes the following form in the time domain:

(10) $$\begin{array}{c} \rho_{t}=\sum_{r_{r}}H_{t}\left(r_{r}\right){⊗}_{t}S_{t}\left(r_{r}\right) \end{array}$$

where $\rho_{t}=F^{-1}\left(\rho \right)$ (and equivalently for $H_{t}$ and $S_{t}$) and where ⊗ stands for the time-domain convolution product that can be substituted with Toeplitz matrices [32]:

(11) $$\begin{array}{c} \rho_{t}=\sum_{r_{r}}T\left(r_{r}\right)S_{t}\left(r_{r}\right) \end{array}$$

with $T\left(r_{r_{i}}\right)\in C^{\;\left(2\;n_{t}-1\right)\times n_{t}}$, the Toeplitz matrix, built from the transfer function $H_{t}\left(r_{r_{i}}\right)$ in the following way:

(12) $$\begin{array}{c} T\left(r_{r_{i}}\right)=\left[\begin{array}{ccccc} H_{t_{1}}\left(r_{r_{i}}\right) & 0 & \ldots & 0 & 0\\ H_{t_{2}}\left(r_{r_{i}}\right) & H_{t_{1}}\left(r_{r_{i}}\right) & \ldots & \vdots & \vdots \\ H_{t_{3}}\left(r_{r_{i}}\right) & H_{t_{2}}\left(r_{r_{i}}\right) & \ldots & 0 & 0\\ \vdots & H_{t_{3}}\left(r_{r_{i}}\right) & \ldots & H_{t_{1}}\left(r_{r_{i}}\right) & 0\\ H_{n_{t}-1}\left(r_{r_{i}}\right) & \vdots & \ldots & H_{t_{2}}\left(r_{r_{i}}\right) & H_{t_{1}}\left(r_{r_{i}}\right)\\ H_{n_{t}}\left(r_{r_{i}}\right) & H_{n_{t}-1}\left(r_{r_{i}}\right) & \vdots & \vdots & H_{t_{2}}\left(r_{r_{i}}\right)\\ 0 & H_{n_{t}}\left(r_{r_{i}}\right) & \ldots & H_{n_{t}-2}\left(r_{r_{i}}\right) & \vdots \\ 0 & 0 & \ldots & H_{n_{t}-1}\left(r_{r_{i}}\right) & H_{n_{t}-2}\left(r_{r_{i}}\right)\\ \vdots & \vdots & \vdots & H_{n_{t}}\left(r_{r_{i}}\right) & H_{n_{t}-1}\left(r_{r_{i}}\right)\\ 0 & 0 & 0 & \ldots & H_{n_{t}}\left(r_{r_{i}}\right) \end{array}\right]. \end{array}$$

The circulant structure of this matrix thus makes it possible to have an algebraic representation of the discrete convolution calculation in the matrix form, which paves the way for the use of pseudo-inversion techniques in the considered application.

The expression obtained in Equation (11) must finally be modified so that it only requires a single matrix operator corresponding to both filtering through all the component transfer functions and the summation operation. It is thus proposed to consider the following formalism:

(13) $$\begin{array}{c} \rho_{t}=T\;\mathrm{vec}\left(S_{t}\right). \end{array}$$

In this final form, the matrix $T\in C^{\;\left(2\;n_{t}-1\right)\times \left(n_{t}.n_{r_{r}}\right)}$ corresponds to the concatenation in the discrete aperture space of all the Toeplitz matrices, so that $T=\left(T\left(r_{r_{1}}\right),\ldots ,T\left(r_{r_{n}}\right)\right)$. In this context, it is now possible to calculate an estimate of *S* from the measured signal:

(14) $$\begin{array}{c} \mathrm{vec}\left({\hat{S}}_{t}\right)=T^{+}\rho_{t}. \end{array}$$

**Figure 4:**
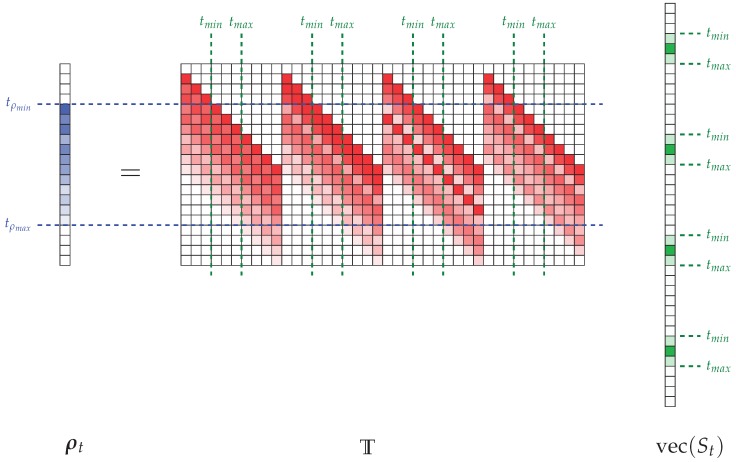
Illustration of the matrix formatting of the relationship between $\rho_{t}$ and $\mathrm{vec}(S_{t})$ for a system with four radiating elements. The number of time samples is deliberately chosen small to facilitate the representation. Time boundaries are identified according to the two dimensions of matrix T, limiting the dimensions of the calculation by removing zero contributions, which represent a significant burden on the total volume of the calculation.

Under these conditions, it may seem useful to reduce the dimensions of the problem by exploiting the sparsity of the time-dependent signals, truncating the matrix T according to its two dimensions (Figure 4).

**Figure 5:**
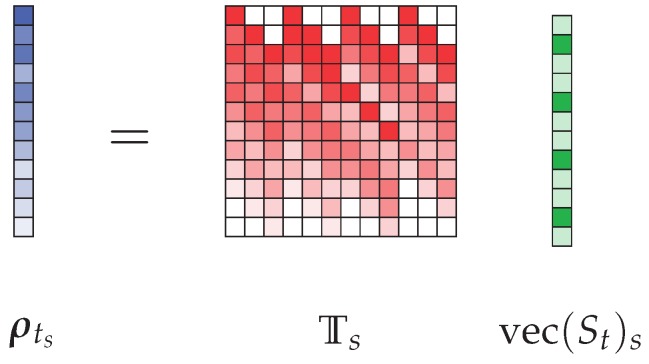
Representation of the compact form of the matrix relation between $\rho_{t_{s}}$ and $\mathrm{vec}{\left(S_{t}\right)}_{s}$, truncated thanks to the sparse properties of the time-domain signals studied in the context of short-range imaging applications.

A first truncation of T is possible in the dimension $\left(n_{t}.n_{r_{r}}\right)$ corresponding to the vectorization of $S_{t}$. For this purpose, it is necessary to determine the minimum and maximum times, respectively $t_{min}$ and $t_{max}$, where the signal is non-zero. This evaluation can be carried out using for example optical imaging systems, converting distance information into equivalent flight time, or directly using a first reconstruction with the equalization method as in the proposed studies. It is not necessary to be highly precise in this evaluation to converge towards an acceptable reconstruction of the signals received by the antennas, but the optimization of the selection of non-zero time domain samples significantly reduces the size of the final matrix to inverse. It is also possible to reduce the second dimension of T by identifying in $\rho_{t}$ the minimum and maximum boundaries of the measured signal, respectively, called $t_{\rho_{min}}$ and $t_{\rho_{max}}$. Finally, if the interval $\left[t_{min},t_{max}\right]$ is defined on $n_{s}$ time-domain samples and if the interval $\left[t_{\rho_{min}},t_{\rho_{max}}\right]$ corresponds to $n_{\rho }$ samples, it is possible to reduce the matrix T to a smaller matrix $T_{s}\in C^{\;n_{\rho }\times n_{s}.n_{r_{r}}}$. The vectors corresponding to each dimension are also truncated so as to obtain $\rho_{t_{s}}\in C^{\;n_{\rho }\times 1}$ and $\mathrm{vec}{\left(S_{t}\right)}_{s}\in C^{\;n_{s}.n_{r_{r}}\times 1}$. A graphical representation corresponding to the reduced form of the problem shown in Figure 4 is provided in Figure 5.

In this reduced form, it is possible to identify that the only remaining parts of the initial matrix correspond to the intersection of the identified time boundaries on the $\rho_{t}$ and $\mathrm{vec}(S_{t})$. This approach thus makes it possible to give a new compact representation of the relation between the measured signal and the signals received in the radiating aperture by identifying and then removing as many elements as possible that do not contribute to this problem.

This double time-domain truncation finally makes it possible to express a more compact relationship between the measured signal and the signals in the radiating aperture:

(15) $$\begin{array}{c} \rho_{t_{s}}=T_{s}\mathrm{vec}{\left(S_{t}\right)}_{s} \end{array}$$

allowing now for the computation of an approximation of the signals received by the radiating elements $\mathrm{vec}{\left({\hat{S}}_{t}\right)}_{s}$ by means of direct or iterative pseudo-inversion techniques:

(16) $$\begin{array}{c} \mathrm{vec}{\left({\hat{S}}_{t}\right)}_{s}=T_{s}^{+}\rho_{t_{s}} \end{array}$$

**Figure 6:**
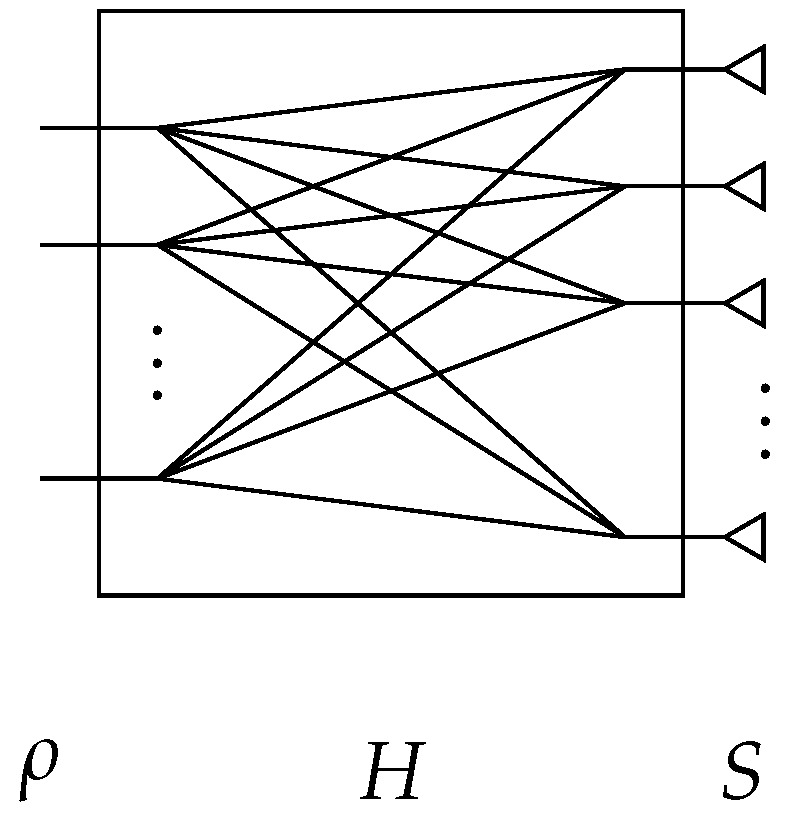
Illustration of a computational imaging component with several measurement ports. The increase in the number of measured samples allows for a more accurate reconstruction of the signals in the plane of the radiating aperture, especially when the number of antennas is large.

An interesting property of circulant matrices simplifies the calculation of $T_{s}$, especially when signals are acquired using frequency measurement tools, as they can be diagonalized using Fourier basis. The compact sensing matrix $T_{s}=\left(T_{s}\left(r_{r_{1}}\right),\ldots ,T_{s}\left(r_{r_{n}}\right)\right)$ is obtained from the concatenation of truncated Toeplitz matrices computed as follows:

(17) $$T_{s}\left(r_{r_{i}}\right)=D_{\left[t_{min},t_{max}\right]}\;\mathrm{diag}\left(H\left(r_{r_{i}}\right)\right)\;D_{\left[t_{\rho_{min}},t_{\rho_{max}}\right]}^{†}\;$$

where $H(r_{r_{i}})$ is a frequency-dependent eigenvector of $n_{\omega }$ samples corresponding to the transfer function of the computational imaging component measured at the location $r_{r_{i}}$ of the radiating aperture and the couple of matrices $D_{\left[t_{\rho_{min}},t_{\rho_{max}}\right]}\in C^{\;n_{\rho }\times n_{\omega }}$ and $D_{\left[t_{min},t_{max}\right]}\in C^{\;n_{s}\times n_{\omega }}$ are discrete Fourier transform matrices computed in the bounds of the time-domain values specified in the index. In this way, it is not necessary to calculate and then truncate the entirety of the sensing matrix T, limiting memory consumption by directly estimating its compact form $T_{s}$. This technique can finally be extended to the more general case of a component with several measurement ports. The implementation of such a system remains justified insofar as the number of measured signals remains lower than the number of antennas (Figure 6).

In such a system where $m_{\rho }$ measurement ports are connected to the computational system, the dimensions of the measured signal matrix then become $\rho_{t}\in C^{\;m_{\rho }\times n_{t}}$ and the transfer function matrix is also $m_{\rho }$ times larger, such as $H\in C^{\;m_{\rho }\times n_{\omega }\times n_{r_{r}}}$. It is possible to keep exactly the same formalism as before by using a simple vectorization of the matrix of measured signals, which in its compact form becomes ${\rho_{t}}_{s}\in C^{\;m_{\rho }\times n_{\rho }}$, adapting the dimensions of the compact sensing matrix, which then takes the following dimensions $T_{s}\in C^{\;m_{\rho }.n_{\rho }\times n_{s}.n_{r_{r}}}$, where, as a reminder, $m_{\rho }$ corresponds to the number of measurement ports, $n_{\rho }$ to the number of time-domain samples of the measured signals after truncation, $n_{s}$ to the number of time-domain samples of the signals on the antennas after truncation, and $n_{r_{r}}$ to the number of radiating elements connected to the computational imaging system. In such conditions, the link between measured and antenna signals is expressed as follows:

(18) $$\begin{array}{c} \mathrm{vec}{\left(\rho_{t}\right)}_{s}=T_{s}\mathrm{vec}{\left(S_{t}\right)}_{s}. \end{array}$$

The quality of the reconstruction will directly depend on the properties and dimensions of the sensing matrix $T_{s}$, which can be studied through a decomposition into singular values to determine the degree of correlation between its different rows and columns and the number of unknowns that can be reconstructed [33,34]. The effectiveness of the proposed technique depends directly on the estimation of the time bounds within which the received signals are defined. The use of such properties for signal estimation has interesting links with the principle of support detection used in the reconstruction of sparse signals [35], notably considered in magnetic resonance imaging [36].

Now that the theoretical elements necessary for understanding this new technique have been presented, it is necessary to validate its functionality. A first numerical simulation of computational imaging is thus conducted in the next section in order to compare the quality and reconstruction time of various image reconstruction methods.

## 3. Numerical Validation

**Figure 7:**
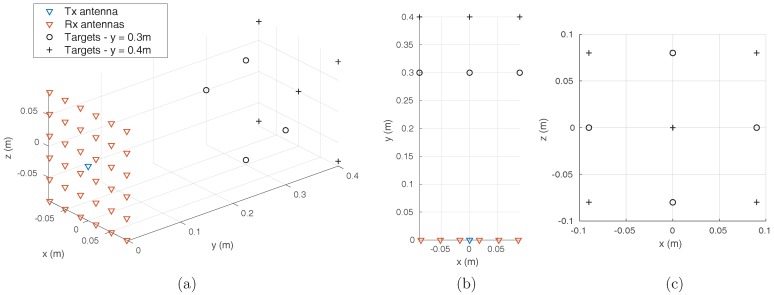
Side (**a**), top (**b**) and transverse (**c**) views of the simulated radar experiment. All receiving antennas are connected to an unrepresented component multiplexing waveforms into a single signal.

A numerical simulation is carried out in the 2–10 GHz band sampled with 1000 frequency points. A radar imaging system made of a transmitting antenna located in the center of a square reception array composed of 36 antennas separated by $0.7\lambda_{c}$ = 3.5 cm according to the two transverse dimensions *x* and *z*, $\lambda_{c}$ being the central wavelength of the operating bandwidth (Figure 7), is proposed.

**Figure 8:**
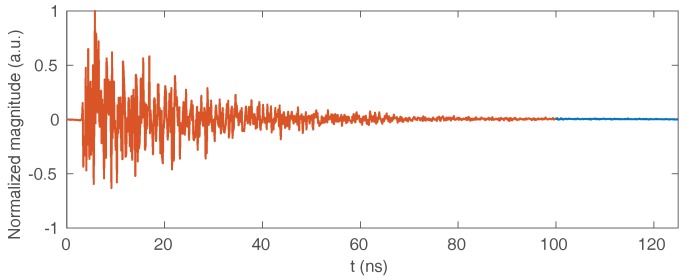
Time signal measured on the single port of the computational imaging system. All 36 signals received by the antennas are encoded by propagation in a passive component whose transfer functions are known. The proposed technique allows for a more accurate estimation of these signals from this single measurement for reconstructing a three-dimensional image. The red line corresponds to the time gating performed on the signal in order to limit the size of the final matrix $T_{s}$.

A set of nine target points are arranged in front of the array in order to be located by the imager. The 36 signals collected by the receiving antennas are then numerically multiplexed to reproduce the behavior of a dispersive component (for example, a cavity) connected to the array. A signal is then measured on its unique output port, from which it is possible to calculate a radar image using the computational approaches presented previously. The multiplexer component is characterized by a quality factor determining the decay time of its time responses, which one wishes the longest possible to limit the level of correlation between its different channels. In the simulated case, the quality factor considered is Q = 377, corresponding to a decay time τ=10 ns. In practice, these values are close to the performance of two-dimensional cavities made with microwave substrates, and are well below the decay times achievable with empty metal cavities whose quality factor easily reaches several thousand. The time-dependent signal measured at the output of the simulated multiplexer component is shown in Figure 8.

**Figure 9:**
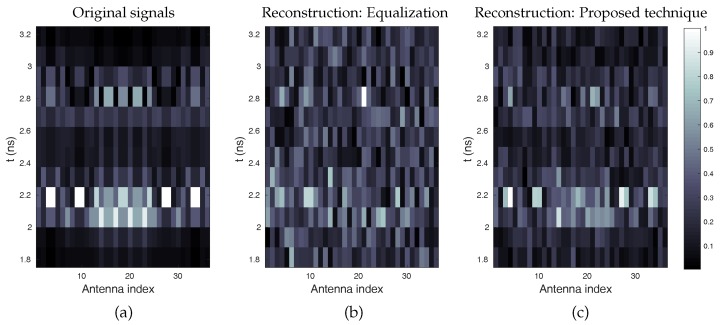
Representation of the normalized magnitude of the time signals received by the antennas. The reconstruction achieved using the proposed technique (**c**) is compared to that obtained in the case of the equalization technique (**b**) described in [12]. The original signals are given for comparison (**a**).

The signal reconstruction is carried out using Equation (7) for the reference equalization technique and Equation (14) for the proposed method. The reconstruction by the proposed technique is based on an approximate prior knowledge of the range covered by the object to be imaged, which can be refined by means of a series of reconstructions making it possible to reduce the size of the time window considered to improve the reconstruction of the signals. The reconstructed signals are presented in Figure 9, and compared to the original signals received by the antennas.

The quality of the reconstructions is evaluated by computing the peak signal-to-noise ratio (PSNR), considering the signals originally received by the radiating aperture as a reference. In the case of the equalization reconstruction technique, the calculated PSNR is 13.6 dB, while the proposed method yields a PSNR of up to 19.2 dB.

**Figure 10:**
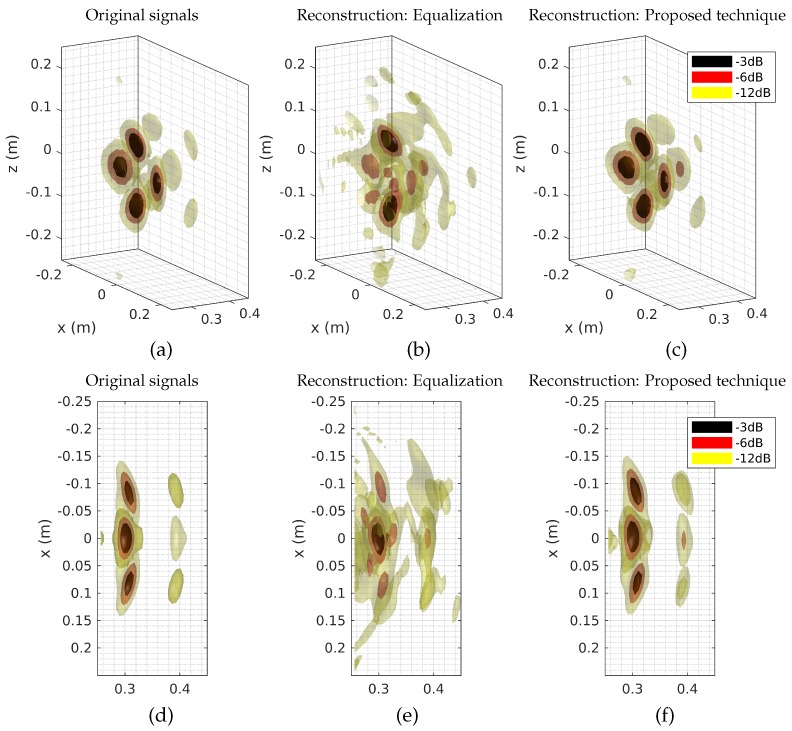
The reconstruction performed from the original signals received by the antennas (side (**a**), top (**d**)) is compared to those obtained in the case of reconstruction by equalization (side (**b**), top (**e**)) and to the proposed technique (side (**c**), top (**f**)). The magnitudes are normalized and displayed as isosurfaces extracted at −3 dB, −6 dB, and −12 dB. Thanks to a better estimation of the signals received compared to the equalization technique initially considered, the proposed technique makes it possible to obtain images closer to those calculated using the original signals, making it possible to find the position of the various targets and limiting the level of speckle present around these.

The proposed technique thus allows for a more accurate reconstruction of the signals received by the antennas compared to the equalization technique initially developed in [12], while remaining compatible with the use of Fourier domain image reconstruction techniques to limit the computation time compared to direct matrix reconstruction as shown in Figure 2 and studied in [23]. In this case, the measured signal time gated between $t_{\rho_{min}}=0$ ns and $t_{\rho_{min}}=100$ ns, and the antenna signal time is truncated between $t_{min}=1.8$ ns and $t_{max}=3.2$ ns, allowing for reconstructions computed on average in 9.5 ms by a generalized minimum residual method after a unique pre-computing time of 11 ms for the evaluation of $T_{s}$, compared to 3 ms with the equalization approach. These calculations are carried out on a computer equipped with a 2.8 GHz dual-core processor. In order to facilitate the interpretation of these results, a reconstruction of the scene is carried out by back-propagation from the signals directly received by the antennas, the signals reconstructed by the equalization technique and by the proposed technique. The results are presented in Figure 10.

Once again, the results corresponding to the proposed reconstruction technique are close to those obtained without the computational approach, in comparison with the image reconstructed using the equalization technique that leads to the creation of artifacts that make the location of the source points difficult. The PSNR is evaluated again to compare these two three-dimensional images with the reference computed with the original signals. The image calculated using equalization corresponds to a PSNR of 19 dB, while the image calculated by the proposed method yields a PSNR of 26.3 dB. The application of such an approach thus enables the reconstruction of an estimate of the target reflection function while decomposing the initial computational imaging problem as expressed by the Equation (2). For an image such as the one presented composed of $n_{r}=50\times 50\times 30=75,000$ voxels, the matrix linking the ρ measure to the f reflection function of the target would have the dimension $M\in C^{n_{\omega }\times n_{r}}$. The use of time-gating on the measured signal ρ can possibly reduce the dimensions of this matrix to $M\in C^{n_{\rho }\times n_{r}}$. In comparison, the proposed technique decomposes this matrix into two parts $T_{s}\in C^{n_{\rho }\times n_{s}.n_{r_{r}}}$ and $G\in C^{n_{s}.n_{r_{r}}\times n_{r}}$, the latter not actually being calculated and replaced by a Fourier domain range migration algorithm. Thus, in the example shown, memory consumption is limited by switching from a matrix $M\in C^{800\times 75,000}$ to a matrix $T_{s}\in C^{800\times 432}$, correspond to a memory consumption 173 times smaller. In an obvious way, the memory consumption and the computation speed is impacted by the depth of the area to be imaged. However, it is possible to reduce the size of the problem even more by also removing the almost zero contributions within the measured signal ρ itself, but such an approach would require the use of non-uniform fast Fourier transforms and is therefore left aside to keep this proof of concept as simple as possible. This selection can be done according to a signal level that may correspond to the noise floor or an arbitrarily higher selected level that will obviously limit the quality of reconstructions of the signals and images but make the overall computation faster and less memory-consuming.

Having presented a numerical validation of this technique that highlights the improvements allowed by this new approach compared to the equalization techniques previously used, it is proposed to validate the application of this technique in an experimental context in the following section.

## 4. Experimental Validation

**Figure 11:**
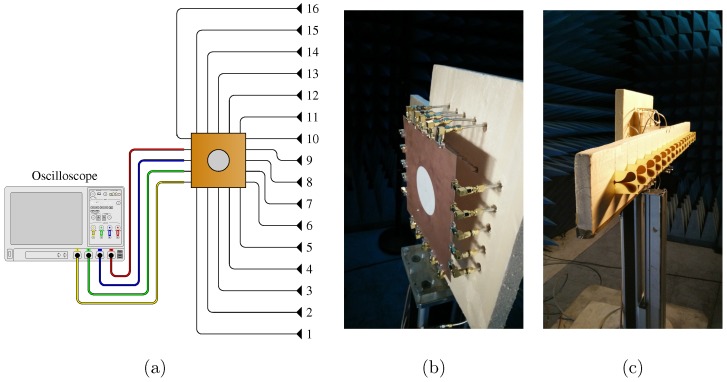
Illustration (**a**) and photographs of the 2D cavity (**b**) connected to both 16 antennas (**c**) and 4 ports of an oscilloscope enabling measurement of a number of signals lower than that of the antennas.

The proposed reconstruction technique is now being confronted with experimental validation in the 2–4 GHz band, using a set-up introduced in [33,34]. The computational imaging component for this application is a $20\times 20\;{\mathrm{cm}}^{2}$ planar cavity with 16 input ports connected to Vivaldi antennas and up to 4 output ports (Figure 11).

**Figure 12:**
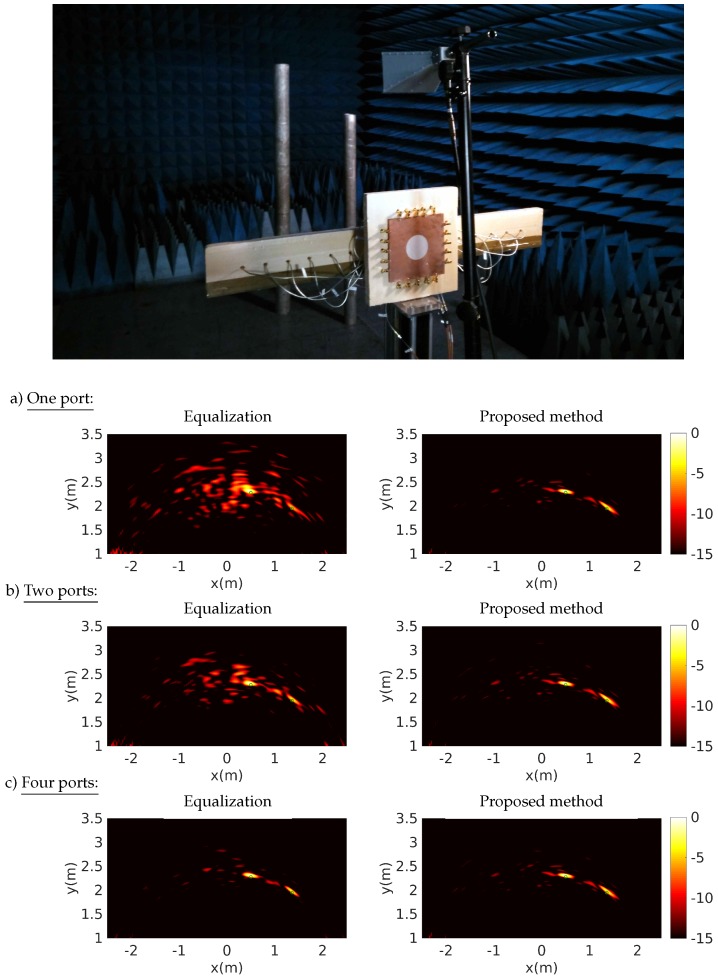
Experimental setup for the localization of two metal cylinders and reconstructions performed by equalization and using the proposed technique with, successively, 1 (**a**), 2 (**b**), and 4 (**c**) signals measured on the computational imaging component ports. The green circles corresponds to the actual locations of the cylinders.

The cavity is fabricated with a 0.65-mm-thick microwave substrate RT/Duroid 6006 with a hole engraved on its top conductor for minimizing the level of correlation between transfer functions [34,37,38]. This experimental demonstration had previously revealed the relationship between the number of signals measured on the ports of a single component and the quality of the reconstructed images, exploiting the frequency diversity of the 4×16 transfer functions measured by 300 frequency samples [33,34]. The average quality factor estimated from the damping time of the impulse responses of this component is about 200. The antenna spacing is 7 cm, which is 0.7 times the central operating wavelength. It is proposed in this framework to compare the reconstruction results obtained with one or several signals measured by the oscilloscope on the output ports of the component, by successively applying the equalization reconstruction technique and the proposed method. The effect of direct coupling is mitigated by performing a blank measurement, subtracted from the acquisitions made when the targets are put in place. Two metal cylinders are arranged opposite the antenna array used in reception and are illuminated by a horn antenna located above the center of the array (Figure 12).

These experiments are conducted using an arbitrary signal generator (Agilent M8190A 12 GSa/s) and oscilloscope (Agilent DSA90404A 20 GSa/s), which yield a maximum frequency of up to 4GHz without the use of mixers. Image computations are performed using back-propagation with the signals reconstructed on the antennas using the reference equalization technique and the proposed method. In this context, the impact of the number of measured signals on the image reconstruction quality is studied using, successively, 1, 2, and 4 responses measured on the 2D cavity ports.

The images are reconstructed from time gated signals between 0 and 30 ns, in each case limiting the appearance of speckle around the targets and, in the proposed method, allowing for a better estimation of the signals received by the antennas. The results obtained using the proposed method seem to present, in each case, a lower level of speckle around the targets and yield a better identification of their position than with the equalization technique. However, it can be seen that the quality of the results obtained with the proposed technique remains limited when using a single signal. In comparison with the numerical study presented above, this experimental demonstration is limited by model approximations and measurement uncertainties that can prevent the algorithm from converging towards an accurate estimation of the signals received by the antennas. However, using more measured information with 2 and 4 signals makes it much easier to converge towards better results.

## 5. Conclusions

A new reconstruction technique adapted to microwave and millimeter-wavecomputational imaging is proposed in this article, taking advantage of the sparsity of signal representation in the time domain. This technique is an interesting alternative to methods based on the use of sensing matrix in computational imaging, which allow for high-quality reconstructions but impose significant levels of memory consumption and computation times. Similar to the equalization techniques initially introduced in this field, the presented approach is based on a decomposition of this sensing matrix in order to reduce its dimensions and make use of conventional Fourier-domain imaging techniques possible. The technique introduced in this article, however, allows more accurate antenna signal estimates to be obtained, while maintaining reasonable computation times that are compatible with real-time applications. After the theoretical concepts necessary for the use of this technique were introduced, the effectiveness of such an approach was validated numerically and then experimentally in the context of depth-limited radar imaging applications that are particularly well suited to this technique.

All time-gating operations presented here are based on the preservation of uniform sampling in order to simplify this proof of principle. The study of very large problems could therefore soon be lightened by adapting this technique to the selection of only measurement samples with a significant amplitude in order to propose reconstructions of comparable quality to the results presented in this article, but calculated in reduced time.
